# Increase in premature mortality due to non-communicable diseases in Sri Lanka during the first decade of the twenty-first century

**DOI:** 10.1186/s12889-018-5503-9

**Published:** 2018-05-02

**Authors:** Dileepa Senajith Ediriweera, Palitha Karunapema, Arunasalam Pathmeswaran, Mahendra Arnold

**Affiliations:** 1 Centre for Health Informatics, Biostatistics and Epidemiology, Faculty of Medicine, University of Kelaniya, Ragama, Sri Lanka; 2 Ministry of Health, Colombo, Sri Lanka; 3 Department of Public Health, Faculty of Medicine, University of Kelaniya, Ragama, Sri Lanka

**Keywords:** Sri Lanka, Non-communicable diseases, Premature non-communicable diseases mortality, Unconditional probability of dying, Global voluntary targets

## Abstract

**Background:**

Globally, non-communicable diseases (NCD) are the leading cause of death and more than 40% of NCD deaths are premature occurring before the age of 70 years. In 2012, World Health Assembly declared its commitment to reduce premature NCD mortality by 25% from 2010 to 2025. The trend of premature NCD deaths in Sri Lanka has not been assessed and thus this study was done to assess it between 2001 to 2010.

**Methods:**

Deaths due to cardiovascular diseases, cancers, chronic respiratory diseases and diabetes were studied. Premature NCD mortality was assessed using unconditional probability of dying (UPoD) due to NCDs among those aged 30 to 70 years. Number of relevant premature NCD deaths that occurred in each 5-year age interval and the respective mid-year population was used to calculate UPoD.

**Results:**

During the period of 2001 to 2010, premature NCD mortality in Sri Lanka increased from 15·8% to 19·1% and males showed higher mortality compared to females throughout the period. Highest mortality was due to cardiovascular diseases followed by cancer and diabetes and all three showed an increasing trend. Chronic respiratory diseases showed an increase until 2004 and dropped thereafter. Among the four NCDs, diabetes revealed the most marked increasing trend in premature mortality during this period.

**Conclusions:**

The data revealed an increasing trend of premature NCD mortality in Sri Lanka between 2001 and 2010 although it has a relatively lower premature NCD mortality rate in the South-East Asian Region. Therefore, reducing premature NCD mortality by 25% from 2010 to 2025 is likely to be a rather challenging task in Sri Lanka and policy level changes need to be taken to achieve this target.

**Electronic supplementary material:**

The online version of this article (10.1186/s12889-018-5503-9) contains supplementary material, which is available to authorized users.

## Background

Globally, non-communicable diseases (NCD) are the leading cause of death accounting for 63% of annual deaths. More than 40% of NCD deaths are premature deaths occurring before the age of 70 and 82% of the premature deaths as well as three quarters of all NCD deaths occur in low-middle countries [1, 2]. At the World Health Assembly held in 2013, much emphasize was placed on reducing premature NCD mortality and a global voluntary target was declared to reduce premature NCD deaths by 25% from 2010 to 2025 [1].

‘Unconditional probability of dying between ages 30 and 70 from selected four NCDs’ (UPoD) is recognized by World Health Organization (WHO) as the progress indicator to monitor reduction in premature NCD mortality. The UPoD indicates the probability of an individual dying between ages 30 to 70 due to the selected four NCDs. This indicator is independent of population age structure and mortality due to other causes. Therefore, it can be used for between country comparisons as well as to track trends of premature NCD mortality. Deaths due to four major NCDs namely; cardiovascular diseases (CVDs), cancers (CAs), chronic respiratory diseases (CRDs) and diabetes (DM) are included in the calculation of the UPoD as these four diseases account for 82% of all NCDs [3, 4]. According to the WHO regional UPoD estimates, South-East Asian Region has the highest UPoD and the Region of the Americas has the lowest UPoD [5] .

Sri Lanka is a lower middle-income South Asian country with a population of 20·8 million people with a per capita income of USD 3912 in 2015 [6] and held the 73rd place in human development index in 2015 [7]. The state healthcare service of Sri Lanka is funded by general tax revenue and covers the entire country. State healthcare services are free at the point of delivery and includes both allopathic and indigenous systems. Allopathic system is considered as the mainstream practice and provides all levels of care (i.e., primary, secondary and tertiary) [8]. The registration of births and deaths are compulsory in Sri Lanka and the Registrar General’s Department of Sri Lanka is responsible for civil registration (i.e. births, marriages and deaths) in the country. Sri Lanka has adopted the International Classification Diseases 10th Revision (ICD-10) for classifying morbidity and mortality [9]. In 2014, crude birth and death rates for Sri Lanka were 16·9 and 6·2 per 1000 population, and the male and female life expectancies were 72·0 and 78·6 years respectively. The leading causes of hospital deaths include ischaemic heart diseases (ICD code: I20 - I25, 30·6 per 100,000), neoplasms (C00 - D48, 24·0 per 100,000), zoonotic and other bacterial diseases (A20 - A49, 18·6 per 100,000). Chronic NCDs are responsible for 71% of all annual deaths in Sri Lanka in 2014 [10].

This analysis was done to determine the recent trend in premature NCD mortality in Sri Lanka with an aim to assess the feasibility of reducing premature NCD mortality by 25% between 2010 and 2025 which is a global voluntary target.

## Methods

We considered the cause of death data of Sri Lanka in 2001, 2002, 2003, 2004, 2005, 2009, 2010 for the analysis. Cause of deaths were separately obtained for males and females in five-year age intervals (e.g. zero to 4 years, five to 9 years). Starting point for the analysis was selected based on the population census 2001. A census was not performed for 20 years before 2001 and thus the population projection data for the period before 2001 was not included in the analysis. Cause of death data for 2006, 2007, 2008 and for the period after 2010 were not available at the time of analysis. Deaths due to NCDs were categorized into four major groups, i.e., cardiovascular diseases (ICD 00–99), neoplasm (ICD C00 – C99), chronic respiratory diseases (J30 – J98) and diabetes (E10 – E14). Similarly, we obtained mid-year population data for population, males and females in five-year age intervals for the period of 2001 to 2010 published by the Census and Statistics Department of Sri Lanka. Population census in 2001 covered only 18 districts out of 25 due to the war in the North and East regions of the country. Therefore, we obtained an estimated midyear population for 2001.

Age standardized death rates (ASMR) were calculated based on the WHO 2000–25 World Standard Population Distribution. Unconditional probability of dying due to NCDs during age 30 to 70 years was determined using the standard formula (Refer Additional file 1 for calculation steps) [1]. Initially, age specific mortality rates and probability of dying for each 5-year age intervals were calculated. Subsequently, probability of dying between age 30 and 70 was calculated. All the calculations were done in R programming language version 3·2·3 [11].

## Results

During the period of 2001 to 2010, mid-year population of Sri Lanka had increased from 18·8 to 20·6 million and the reported total deaths due to four NCDs had increased from 41,808 to 62,565 resulting in an increased NCD related crude death rate from 2·2 to 3·0 per 1000. However, ASMR showed an initial decrease from 2001 to 2002 and thereafter it steadily increased until 2010. Throughout the period under review, a higher crude death rate and ASMR were reported for males compared to females (Table 1).

**Table 1:** Population and non-communicable disease mortality statistics from 2001 to 2010

	Both sexes - all age groups				Males - all age groups				Females - all age groups			
Year	Mid-year population	Total deaths from 4 NCDs^a^	Death rate^a^	ASMR	Mid-year population	Total deaths from 4 NCDs	Death rate	ASMR	Mid-year population	Total deaths from 4 NCDs	Death rate	ASMR
2001	18,797,000^b^	41,808	2.2	2.1	9359,000^b^	25,526	2.7	4.8	9438,000^b^	16,282	1.7	3.7
2002	18,921,000	42,485	2.2	1.4	9,350,000	25,963	2.8	3.7	9,571,000	16,522	1.7	2.1
2003	19,173,083	45,350	2.4	1.5	9,475,000	27,358	2.9	3.9	9,698,083	17,992	1.8	2.3
2004	19,433,360	47,856	2.5	1.6	9,676,883	28,798	3.0	4.0	9,756,478	19,058	1.9	2.4
2005	19,643,474	49,013	2.5	1.6	9,782,497	29,488	3.0	4.0	9,860,977	19,525	1.9	2.4
2009	20,476,000	60,880	3.0	1.8	10,174,000	34,830	3.4	4.4	10,302,000	26,050	2.5	3.0
2010	20,675,000	62,565	3.0	1.8	10,273,000	35,584	3.5	4.4	10,402,000	26,981	2.6	3.0

*ASMR* Age standardized death rates

^a^Death rates per 1000 population ^b^Estimated mid-population (2001 census was done only in 18 districts out of 25)

The 30–70 year age group represented 42·7% (i.e. 8,037,000 / 18,797,000 X 100%) of the total population in 2001 and it was 42·8% (i.e. 8,842,000 / 20,675,000 X 100%) in 2010, representing almost a static population age structure for the 30 to 70 age group during the study period. Overall, the crude death rate due to the four NCDs had increased from 2·8 to 3·4 during the study period, while ASMR failed to show a clear trend from 2001 to 2010. Nevertheless, a higher crude death rate and an ASMR were reported among males compared to females within this age group (Table 2).

**Table 2:** Population and non-communicable disease mortality statistics of the age 30–70 group from 2001 to 2010

	Both sexes 30–70 age groups				Males - 30 – 70 age groups				Females - 30 – 70 age groups			
Year	Mid-year population	Total deaths from 4 NCDs	Death rate^a^	ASMR	Mid-year population	Total deaths from 4 NCDs	Death rate^a^	ASMR	Mid-year population	Total deaths from 4 NCDs	Death rate^a^	ASMR
2001	8037,000^b^	22,864	2.8	3.1	3950,000^b^	14,810	3.7	7.8	4087,000^b^	8054	2.0	4.8
2002	8,070,000	22,779	2.8	1.7	3,927,000	14,870	3.8	4.7	4,143,000	7909	1.9	2.2
2003	8,179,297	23,995	2.9	1.7	3,980,000	15,546	3.9	4.8	4,199,297	8449	2.0	2.3
2004	8,309,920	25,321	3.0	1.8	4,086,766	16,431	4.0	4.9	4,223,153	8890	2.1	2.4
2005	8,399,848	25,410	3.0	1.8	4,132,031	16,307	3.9	4.7	4,267,817	9103	2.1	2.4
2009	8,755,000	29,659	3.4	1.9	4,296,000	18,631	4.3	5.1	4,459,000	11,028	2.5	2.7
2010	8,842,000	29,933	3.4	1.9	4,339,000	18,846	4.3	5.0	4,503,000	11,087	2.5	2.6

*ASMR* Age standardized death rates

^a^Death rates per 1000 population ^b^Estimated mid-population (2001 census was done only in 18 districts out of 25)

**Fig. 1:**
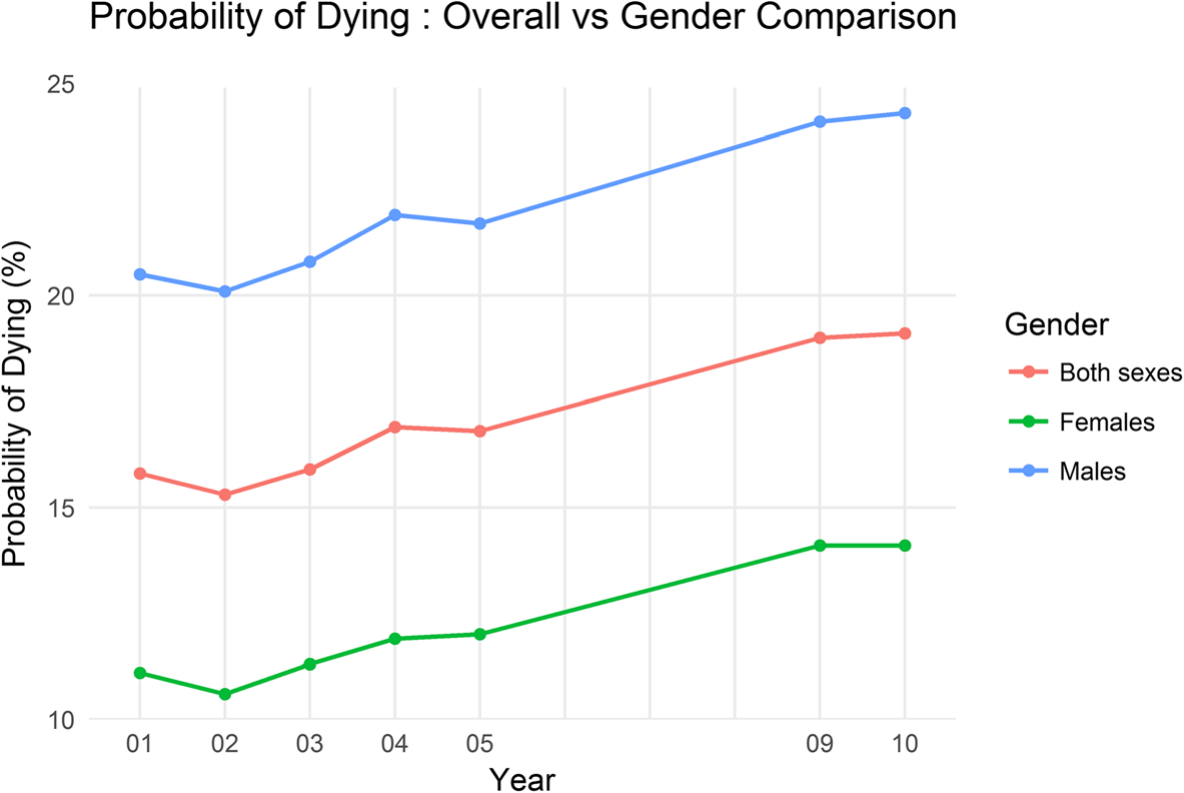
Unconditional probability of dying due to four major non-communicable diseases between ages 30 to 70 from 2001 to 2010 for both sexes, males and females. Unconditional probability of dying has increased during from 2001 to 210. Males are showing higher unconditional probability of dying compared to females

**Fig. 2:**
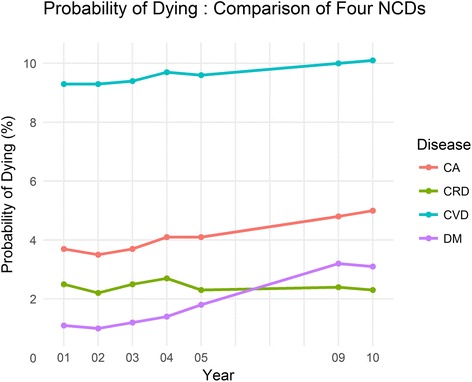
Unconditional probability of dying due to four major non-communicable diseases between ages 30 to 70 from 2001 to 2010 for cardiovascular diseases (CVD), cancer (CA), diabetes mellitus (DM) and chronic respiratory diseases (CRD). CVD shows the highest unconditional probability of dying followed by CA and DM; DM has overridden CRD in recent years

The overall UPoD due to the four NCDs had steadily increased from 15·8% to 19·1% during the period between 2001 and 2010 with a higher UPoD for males compared to females. Deaths related to cardiovascular diseases, diabetes and cancer illustrated an increasing trend throughout the period. The deaths due to chronic respiratory diseases demonstrated an increase until 2004 and it dropped thereafter. Among the four NCDs, diabetes revealed the highest increasing trend in UPoD during the study period. Similarly, a higher UPoD was visible among males compared to females in each of the four major NCDs throughout the study period (Table 3, Figs. 1 and 2).

**Table 3:** Unconditional probability of dying (%) due to four non-communicable diseases between ages 30 to 70 from 2001 to 2010

	All four major Non-communicable Diseases			Cardiovascular Diseases			Diabetes Mellitus			Cancer			Chronic Respiratory Diseases		
Year	Overall	Males	Females	Overall	Males	Females	Overall	Males	Females	Overall	Males	Females	Overall	Males	Females
2001	15.8	20.5	11.1	9.3	13.0	5.7	1.1	1.3	0.9	3.7	4.1	3.3	2.5	3.5	1.6
2002	15.3	20.1	10.6	9.3	13.1	5.7	1.0	1.2	0.8	3.5	4.0	3.1	2.2	3.1	1.3
2003	15.9	20.8	11.3	9.4	13.1	5.9	1.2	1.3	1.0	3.7	4.3	3.2	2.5	3.5	1.6
2004	16.9	21.9	11.9	9.7	13.5	6.0	1.4	1.6	1.2	4.1	4.7	3.6	2.7	3.7	1.7
2005	16.8	21.7	12.0	9.6	13.5	5.9	1.8	2.1	1.5	4.1	4.5	3.6	2.3	3.2	1.5
2009	19.0	24.1	14.1	10.0	13.6	6.5	3.2	3.7	2.7	4.8	5.4	4.2	2.4	3.4	1.5
2010	19.1	24.3	14.1	10.1	13.8	6.5	3.1	3.7	2.6	5.0	5.8	4.3	2.3	3.2	1.4

## Discussion

In Sri Lanka, UPoD due to cardiovascular diseases, cancer, diabetes and chronic respiratory diseases have increased from 15·8 to 19·1 during the period between 2001 and 2010 demonstrating an increasing trend in premature deaths due to NCDs. Therefore, reducing premature mortality due to NCDs by 25% from 2010 to 2025 seems to be a challenging task for Sri Lanka [12] despite having a general tax revenue funded healthcare system covering the entire country [8]. The UPoD of Sri Lanka during this period is similar to the figures of the WHO Region of the Americas and much lower than the WHO estimates for South-East Asian Region [5] as well as the neighboring country India [13]. However, the worrying feature is that the rates have increased during this period in Sri Lanka whereas the rates have decreased in all other WHO regions.

Throughout the study period, 30 to 70 age group showed a higher NCD crude death rate and a higher NCD ASMR compared to the rates of the entire population. Among NCD deaths there were more male deaths for both the entire population and within the 30 to 70 age group. The premature NCD crude death rate as well as the premature NCD ASMR were almost twice as high among males compared to females during this period. This is in line with the higher life expectancy of females in Sri Lanka [10].

The overall UPoD has increased during the period from 2001 to 2010 in Sri Lanka which reflects the loss of a considerable proportion of the workforce in the country. The highest proportion of UPoD was due to cardiovascular diseases; therefore, main preventive and curative measures must be geared towards prevention of premature cardiovascular diseases deaths in Sri Lanka. Individual based health service interventions for high risk individuals and people who have acute cardiovascular diseases are also needed to be undertaken to reduce cardiovascular disease mortality in the country. According to the results, the second highest UPoD was due to cancers during this period. A previous study has also revealed that the incidence of cancers increased from 27·9 to 52·3 in Sri Lanka from 1985 to 2007 [14]. The highest increasing UPoD trend was seen in DM. A similar trend was observed by a previous study that revealed an over 300% increase of diabetes prevalence since late 1980s to 2005 in Sri Lanka [15]. Socio-economic and lifestyle changes could explain such epidemiological transition within a relatively short period of time span. Policy level changes need to be taken to reduce risk factors such as alcohol use, tobacco use, high body mass index, high cholesterol, high blood glucose, low fruit and vegetable intake, and physical inactivity to control NCD mortality [16].

The UPoD due to chronic respiratory diseases had increased slowly until 2004 and remained at a plateau phase until 2010. Government hospital admissions due to respiratory diseases had remained almost static between 2000 and 2007 [17] and this might be due to the improvement in asthma and chronic obstructive pulmonary disease management in Sri Lanka.

Assessing the NCD mortality in Sri Lanka is challenging due to the lack of infrastructure for civil registration, death certification, uncertainty in reporting cause of death, errors in ICD coding as well as under reporting and lack of timeliness. It is also difficult to determine mortality due to specific NCD, as NCDs are often related to each other and changing population structure also needs to be considered when comparing mortality rates over a period. Therefore, we used UPoD methodology which is the WHO recommended indicator to compare the mortality data within a country in different time periods. The relatively poor quality of civil registration data has been addressed by inclusion of broad NCD disease categories.

Although Sri Lanka faced a war during the period in which data were recorded, it is unlikely that it affected the results of our study. The war started in 1983 and a considerable outmigration took place to non-war areas during the early years of the war. Therefore, the majority of total NCDs mortality data was captured by the Registrar General’s Department. It is also unlikely that outmigration contributed to increase in NCD mortality during the study period as outmigration was restricted in late 90s. It is unlikely that the rising trend of UPoD towards 2010 happened due to improved mortality reporting. The war ended in 2009 and it took several years for the resettlement to take place and to build medical infrastructure to have an impact on mortality reporting.

There are several limitations in this study. We could not obtain data beyond 2010 from Register General’s Department due to data unavailability at the time of analysis. We used the national data on cause of death for Sri Lanka by the Registrar General’s Department; however, underreporting may have influenced the results. Mortality data was also not available for 2006 to 2008 and could not be included in the analysis. There could be a possibility of overestimation of UPoD at later years due to the improvement in death reporting in the country, improved classification of cause of death and ICD classification. A higher UPoD in both sexes, males, females, DM, cancer and COPD was noticed in 2001 compared to 2002. This could be attributed to the possible underestimated mid-year population in 2001 calculated based on 18 out of 25 districts in the country.

## Conclusions

Sri Lanka faces NCD epidemic in terms of high mortality and morbidity. This is clearly shown by the rising trend of crude death rates, ASMR and UPoD due to four major NCDs during the first decade of the twenty-first Century. The majority of NCD deaths occur within the workforce in the country and that negatively influences country’s economy. Therefore, policy level changes need to be taken to prevent and control NCDs and ensure effective implementation of programmes. Sri Lanka is unlikely to achieve a 25% reduction in premature NCD mortality by 2025 if the present increasing trend is not reversed. The UPoD due to NCDs must be monitored closely with improved quality of data with a view of providing solid evidence to policy makers.

## Additional file

Additional file 1: Annexure. (DOCX 33 kb)

## Abbreviations

ASMR: Age standardized death rates
CAs: Cancers
CRDs: Chronic respiratory diseases
CVDs: Cardiovascular diseases
DM: Diabetes
NCD: Non-communicable diseases
UPoD: Unconditional probability of dying between ages 30 and 70 from selected four NCDs
WHO: World Health Organization

## References

[1] Mendis S (2014). Global status report on noncommunicable diseases 2014.

[2] World Health Organization (2015). Noncommunicable diseases.

[3] Hunter DJ, Reddy KS (2013). Noncommunicable diseases. N Engl J Med.

[4] NCD global monitoring framework. Geneva: World Health Organization; 2013.

[5] World Health Statistics 2016. Monitoring health for sustainable development goals. Geneva: World Health Organization; 2016.

[6] Sri Lanka Overview. World Bank; 2016. http://www.worldbank.org. Accessed Jan 23 2017.

[7] Human Development Report 2015. New York: United Nations Development Program; 2015.

[8] Annual report 2012. Colombo: Central Bank of Sri Lanka; 2012.

[9] Gamage S, Rampatige R, Samarakoon J, Ranadheera S, Mikkelsen L, Aung E (2009). Assessing the production, quality and use of national vital statistics: a case study of Sri Lanka.

[10] Annual health bulletin 2014. Colombo: Ministry of Health; 2016.

[11] R Core Team (2015). R: a language and environment for statistical computing.

[12] A comprehensive global monitoring framework including indicators and a set of voluntary global targets for the prevention and control of noncommunicable diseases. Geneva: World Health Organization; 2012.

[13] Noncommunicable diseases country profiles 2014. Geneva: World Health Organization; 2014.

[14] Cancer Incidence Data 2009. Colombo: National Cancer Control Program; 2015.

[15] Jayawardena R, Ranasinghe P, Byrne NM, Soares MJ, Katulanda P, Hills AP (2012). Prevalence and trends of the diabetes epidemic in South Asia: a systematic review and meta-analysis. BMC Public Health.

[16] Stevens G, Mascarenhas M, Mathers C (2009). Global health risks: progress and challenges. Bull World Health Organ.

[17] Non-communicable disease statistics - 2007. Hospital discharge data. Colombo: Ministry of Health; 2011.

