# Supplementary material for: Increase in premature mortality due to non-communicable diseases in Sri Lanka during the first decade of the twenty-first century
Source: BMC Public Health. 2018 May 2;18:584. doi: 10.1186/s12889-018-5503-9 (PMC5932898; doi:10.1186/s12889-018-5503-9)
Supplement: Supplementary file 1 — Annexure. (DOCX 33 kb) [file 12889_2018_5503_MOESM1_ESM.docx]

**Annexure**

Step 1: Age-specific mortality rate from four NCDs

$${}_{5}M_{x}=\frac{{}_{5}D_{x} from 4 NCDs}{{}_{5}P_{x}}$$

Step 2: Probability of dying in each five-year age range

$${}_{5}q_{x}=\frac{5 \times{}_{5}M_{x}}{1 + 2.5 \times{}_{5}M_{x}}$$

Step 3: Probability of dying during age 30 to 70 (${}_{40}q_{30}$)

$${}_{40}q_{30}=1-\left(1-{}_{5}q_{30}\right)\times\left(1-{}_{5}q_{35}\right)\times\ldots\left(1-{}_{5}q_{65}\right)$$

Where;

$x=$ exact age in years.

$n=$ length of interval in years. This symbol is omitted when$n=1$.

${}_{n}D_{x}=$ number of deaths occurring to persons aged$x$ to $x+n$of the period under consideration.

${}_{n}P_{x}=$ number of persons aged$x$ to $x+n$ alive at the mid-point of the period under consideration.

${}_{n}M_{x}=$age-specific death rates calculated from information on deaths among persons aged$x$ to $x+n$during a given year and the population aged $x$ to $x+n$at the mid-point of the same year.

${}_{n}q_{x}=$ Probability of dying between exact ages$x$and$x+n$.
